# Parenthood does not explain the gender difference in clinical position in academic medicine among Swedish, Dutch and Austrian physicians

**DOI:** 10.1007/s10459-019-09882-9

**Published:** 2019-03-06

**Authors:** Nikola Komlenac, Marie Gustafsson Sendén, Petra Verdonk, Margarethe Hochleitner, Heidi Siller

**Affiliations:** 1 Gender Medicine Unit, Medical University of Innsbruck, Innrain 66, 6020 Innsbruck, Austria; 2 Department of Psychology, Stockholm University, 106 91 Stockholm, Sweden; 3 Department of Social Sciences, Södertörn University, 106 91 Stockholm, Sweden; 4 APH research institute, Amsterdam UMC-VUmc, Boelelaan 1089a, 1081 HV Amsterdam, The Netherlands

**Keywords:** HOUPE II, Physicians, Academic career, Parenthood, Publication activity, Sweden, Austria, The Netherlands

## Abstract

Studies have continuously shown that fewer women than men achieve leadership positions in academic medicine. In the current study we explored gender differences in clinical position among academic physicians at three university hospitals, each in a different European country. These countries, Sweden, the Netherlands and Austria, differ in terms of gender equality. We analyzed whether the number of children, working hours or publications could explain gender differences in physicians’ clinical position. In this cross-sectional questionnaire study 1333 (54% female) physicians participated. Physicians were asked about their gender, age, number of children, working hours and clinical position. We used structural equation models to explore the influence of gender on the physicians’ clinical position in each of the three countries. We explored whether the association between gender and clinical position could be explained by number of children, working hours or publication activity. The analyses revealed that at all three university hospitals gender influenced clinical position. These gender differences in clinical position could be partly explained by gender differences in publication activity. Female physicians as compared to male physicians were likely to publish fewer articles, and in turn these lower publication numbers were associated with lower clinical positions. The number of children or working hours did not explain gender differences in publication activity or clinical position. Therefore, factors other than unequal allocation of household labor, such as the academic working environment, may still disproportionately disadvantage women’s progress, even at universities in countries with high rates of gender equality such as Sweden.

## Introduction

Although the number of women entering and graduating from academic medicine has increased in the past years and reached parity with the number of men, women remain underrepresented at higher levels of academic administration and leadership positions such as department chair or dean (Eloy et al. 2013; Hofler et al. 2016; Lautenberger et al. 2014; Reed et al. 2011; Wehner et al. 2015; White et al. 2012). One conceptual framework often used to explain this imbalance in career progression among academic physicians is the “leaky pipeline” (Ahmad 2017; Etzkowitz et al. 2000; Goulden et al. 2011; National Research Council 1983), which cites starting a family and childbirth as two common factors influencing whether women stay on the academic track or leave it (e.g. Goulden et al. 2011). For female physicians it is apparent that the time of family formation and childbearing conflicts with career progression (Carr et al. 2015; Gonzalez Ramos et al. 2015; Joels and Mason 2014). Different traditional gender role norms for women and men typically ascribe a larger share of household labor to women than men (Berk 2012) and thus may explain why women are more affected by family formation and childbearing in their careers.

### Linear job advancement and family formation in academic medicine

Job advancement in academic medicine follows the norm of linear career development. Academic physicians advance in their career by completing certain faculty stages within a particular time frame. An academic post usually requires the achievement of a doctoral degree. The usual progression of an academic career entails an initial period at assistant or associate level as university faculty and is then followed by a full professorship (Etzkowitz et al. 2000; Wolfinger et al. 2009). Fundamental to this linear job progression is a steadily rising number of publications (Leykum et al. 2011).

However, women face certain disadvantages when attempting to master this linear job progression. They are more likely to develop non-linear job advancement than are men. Family milestones are important factors that negatively influence women’s job advancement and possibly hinder them in achieving linear career development (Gonzalez Ramos et al. 2015).

There is empirical evidence to support the fact that women’s career progress in academic medicine is more negatively affected by starting a family than is men’s career progress. For example, in a study conducted in Switzerland, female physicians with young children were more likely to work part-time and have partners working full-time instead of part-time than were male physicians with young children (Stamm and Buddeberg-Fischer 2011). In another study in the US female academic physicians tended to spend less time on research than did partnered male physicians with children (Jolly et al. 2014).

One reason why women are more affected by childbearing than men and why an unequal allocation of household labor exists among heterosexual couples is that traditional gender role norms ascribe childrearing responsibilities and household chores to the female gender role (Berk 2012). Again, empirical studies support the standpoint that the division of household labor among heterosexual couples with children is unequal. For example, one study in the US with early-career physician-researchers found that among partnered or married physicians with children, female physicians reported spending up to 8.5 h per week more on household chores or parenting than did male physicians (Jolly et al. 2014).

This unequal allocation of household labor makes it more challenging for women than for men to find time for research activities. Therefore, family formation, childbearing and parenthood can conflict with publication productivity (Hunter and Leahey 2010; Stack 2004). Both women and men reported lower publication productivity after childbearing. However, women were affected more than men (Hunter and Leahey 2010).

This disadvantage could be one major factor in the prominent gender difference reported in publication activity worldwide. It is often reported that female physicians publish less often than do male physicians (Burden et al. 2015; Eloy et al. 2013; Jagsi et al. 2006; Jena et al. 2015; Løvseth et al. 2014; Piper et al. 2016; Reed et al. 2011), especially in journals with high impact factors (Lerchenmüller et al. 2018).

### Traditional gender roles influence job advancement

Feminine and masculine traditional gender roles are defined as being in opposition to each other. Traditional gender roles ascribe certain characteristics, attributes or behavioral expectations to a group of people with a certain sex (Connell 2015). Traditional masculine gender roles include attributes such as heroism, competitiveness, rationalism, intellect, distance or objectification. Attributes ascribed to the traditional feminine gender role are characteristics such as cooperation, communication, tenderness or empathy. Characteristics assigned to leadership roles, or researcher roles, are more often congruent with those that define a more traditional masculine gender role and are considered dominant cultural forms of leadership, whereas more feminine characteristics such as admitting to vulnerability or cooperation are less in line with the role of a leader (Bleakley 2013; Savigny 2014).

According to the Role Congruity Theory of Prejudice Towards Female Leaders (Eagly and Karau 2002) the incongruency between traditional feminine gender role norms and traditional leadership roles or the roles of being a researcher markedly disadvantages women’s progression in the workplace. On the one hand, women are not expected to have or display attributes and behaviors that are attributed to the masculine gender role. On the other hand, when women do show such “masculine” “leadership” attributes or behaviors they violate gender role expectations, for which they may be unfavorably judged (Eagly and Karau 2002). For instance, “Women in positions of authority are thought [to be] too aggressive […], and what appears assertive, self-confident, or entrepreneurial in a man often looks abrasive, arrogant, or self-promoting in a woman” (Ely et al. 2011, p. 477). This incongruency between traditional feminine gender role norms and traditional leadership roles disadvantages women’s progress towards leadership positions. It leads to prejudice and discrimination towards women in academia and leadership positions and undermines their credibility as leaders or researchers (Nash and Moore 2018).

In addition, researcher roles are more often congruent with those that define a more traditional masculine gender role. This became evident in an online study in which women, but not men, were perceived to be lacking the requisite qualities for a successful scientist (Carli et al. 2016). Such stereotypes and other prejudices negatively influence women’s work in academia and yet simultaneously privilege men (Sattari and Sandefur 2018).

Most strikingly, these stereotypes and prejudices become evident when people judge researcher’s work, namely grant applications or scientific texts. It was found that people judge identical scientific texts differently solely on the basis of the author’s supposed gender (Goldberg 1968; Handley et al. 2015). People judge applications more negatively when they think the applicant is a woman rather than a man (Moss-Racusin et al. 2012). This gender bias is also evident in grant applications for natural science research funding. A recent Canadian analysis of grant applications submitted to the Canadian Institutes of Health Research showed that female applicants overall rated lower than did male applicants (Tamblyn et al. 2018). In a 1997 groundbreaking Swedish study of the peer review process for the Swedish Medical Research Council, female applicants were seen to need to be 2.5 times as productive as male applicants to receive the same evaluation on their scientific competence (Wennerås and Wold 1997). A recent study showed that, to date, on average women benefit less from the same number of publications than do men when applying for funding or grants (Lerchenmueller and Sorenson 2018).

Traditional gender roles and the stereotypes and other prejudices that are derived from these gender roles can negatively influence women’s working environment in academia because academic medicine is a “gendered profession” (Riska 2008, p. 14) that is still based mostly on masculine values (Bleakley 2013; Riska 2008). Academic medicine can still be described as an “exaggerated old boys’ club” (Pololi and Jones 2010, p. 441). In such environments institutional male homosociality is likely to take place. Homosociality refers to the preference of people to interact or form relationships with members of the same gender. In the case of academic medicine “old boys’ clubs” form (Holgersson 2013; Pololi and Jones 2010, p. 441) and under such conditions women are often excluded from information and informational networks (Sattari and Sandefur 2018) and may feel like “cultural outsiders” (Pololi and Jones 2010, p. 441; Savigny 2014). They may therefore have a poor sense of belonging to their workplace in academic medicine (Pololi et al. 2013). Addressing any gender issues at the workplace additionally “puts women at risk of ‘making excuses’ or playing the ‘victim’ in the eyes of male colleagues” (Nash and Moore 2018, p. 8). A (toxic) working environment that is filled with exclusion, discrimination or prejudice may be one reason why women choose to leave an academic career path and more often choose a career in private practice or choose to pursue a job in another medicine-related field (Riska 2011).

### Differences among European countries

European countries differ widely in terms of gender equality (World Economic Forum 2015). In an overall ranking of gender equality in terms of economic participation, educational attainment or political empowerment of women and men, Sweden was ranked 5th out of 145 countries considered. Thus, in Sweden in many domains, e.g. earned income, proportion of women as legislators, senior officials and managers, gender equality was evident. The Netherlands was ranked 13th in this country comparison, while Austria was considered to offer less gender equality and ranked 37th (World Economic Forum 2015). The fact that Sweden ranked highest in gender equality among these three countries in this comparison was supported by the Gender Equality Index 2017. The Gender Equality Index is a further indicator of gender equality in seven domains: work, money, knowledge, time, power, health and violence (European Institute for Gender Equality 2017).

These countries also differed regarding the percentage of women conducting research in medicine (European Commission 2015). In Sweden the proportion of female researchers in medical sciences was 59%, whereas in the Netherlands and Austria the proportion of female researchers in this field constituted 41% and 46%, respectively. In addition, in Sweden as many heads of higher education institutions were women as were men (50%), whereas in Austria and the Netherlands male heads of higher education institutions made up the majority. In Austria 24% of higher education institution heads were women and in the Netherlands 14% of people holding this position were women (European Commission 2015).

### Aim of the study

The current study adds to the existing literature on gender differences in academic medicine by combining factors of family formation, working hours, publication activity and clinical position in one single study. We explored gender differences in clinical position among academic physicians at three university hospitals in different European countries: Sweden, the Netherlands and Austria. We analyzed whether the number of children, working hours or publication activity could explain gender differences in physicians’ clinical position. We also examined the direct influence of gender on every variable included in the analysis while controlling for the influence of the other factors described.

Because of the high gender equality prevalent in Sweden (World Economic Forum 2015), we speculated that the effect of gender on clinical position, family formation, working time and publication activity would be lowest at the Swedish university hospital. Compared to the Swedish university hospital, we expected these associations between gender and the mentioned factors to be higher at the Dutch and Austrian university hospitals, in that order.

## Materials and methods

### Participants

In total 1367 physicians from a university hospital in Austria (*N* = 111; response rate = 22%), a university hospital in the Netherlands (*N* = 207; response rate = 29%) and a university hospital in Sweden (*N* = 1049; response rate = 39%) participated in the current study. Of these physicians, 34 (2.5%) participants were excluded from the analysis as they did not indicate their gender. After the exclusion 1333 participants remained for the statistical analyses (111 Austrian physicians, 204 Dutch, 1018 Swedish). More than half of the participants (54%) were women, whereby the percentage of women in the respective sub-samples ranged from 40 (Austrian) to 60% (Netherlands; Tables 1, 2, 3).

**Table 1:** Demographic characteristics and gender differences in the Austrian subsample *N* = 111

Characteristic	Value	Men (%)	Women (%)	Gender differences: test statistics
Number of respondents^c^		60%	40%	
Age^b^	< 35	30%	39%	
	35–45	28%	30%	
	45–55	28%	25%	
	55–65	13%	7%	
	> 65	0%	0%	χ^2^(7) = 2.8, *p* = .927
Number of children^b^	None	36%	61%	
	One	16%	21%	
	Two	31%	16%	
	More than two	17%	2%	χ^2^(4) = 11.4, *p* = .016
Working hours as physician	Mean (*SD*)	54 (19)	43 (18)	*t*(109) = 2.9, *p* = .004
Publication activity^b^	None	15%	11%	
	1–5	18%	36%	
	6–15	6%	21%	
	16–30	9%	11%	
	More than 30	52%	20%	χ^2^(6) = 21.3, *p* = .001
Clinical position^b^ (*n* = 99^a^)	Resident	25%	42%	
	Specialist	46%	45%	
	Chief physician	30%	13%	χ^2^(2) = 4.9, *p* = .090

Reported are percentages of each category

^a^Not all totals reflect the full number of participants (*N* = 111) because of missing entries

^b^Percentages refer to the number of responses within the given gender

^c^Percentages refer to the number of responses within the given country

**Table 2:** Demographic characteristics and gender differences in the Dutch subsample *N* = 204

Characteristic	Value	Men (%)	Women (%)	Gender differences: test statistics
Number of respondents^c^		40%	60%	
Age^b^ (*n* = 186^a^)	< 35	22%	53%	
	35–45	40%	35%	
	45–55	24%	9%	
	55–65	13%	3%	
	> 65	0%	0%	χ^2^(7) = 28.2, *p* < .001
Number of children^b^	None	32%	59%	
	One	16%	10%	
	Two	28%	19%	
	More than two	24%	12%	χ^2^(4) = 18.1, *p* < .001
Working hours as physician	Mean (*SD*)	46 (13)	41 (13)	*t*(169.1) = 2.6, *p* = .011
Publication activity^b^ (*n* = 203^a^)	None	7%	22%	
	1–5	26%	45%	
	6–15	26%	22%	
	16–30	12%	8%	
	More than 30	29%	3%	χ^2^(6) = 37.8, *p* < .001
Clinical position^b^ (*n* = 200^a^)	Resident	29%	59%	
	Specialist	50%	36%	
	Chief physician	21%	4%	χ^2^(2) = 23.5, *p *< .001

Reported are percentages of each category

^a^Not all totals reflect the full number of participants (*N* = 204) because of missing entries

^b^Percentages refer to the number of responses within the given gender

^c^Percentages refer to the number of responses within the given country

**Table 3:** Demographic characteristics and gender differences in the Swedish subsample *N* = 1018

Characteristic	Value	Men (%)	Women (%)	Gender differences: test statistics
Number of respondents^c^		46%	54%	
Age^b^	< 35	16%	23%	
	35–45	30%	36%	
	45–55	29%	23%	
	55–65	22%	17%	
	> 65	3%	2%	χ^2^(7) = 18.5, *p* = .016
Number of children^b^ (*n* = 1016^a^)	None	21%	26%	
	One	13%	16%	
	Two	34%	35%	
	More than two	31%	23%	χ^2^(4) = 10.8, *p* = .029
Working hours as physician	Mean (*SD*)	39 (12)	39 (11)	*t*(1016) = − .1, *p* = .918
Publication activity^b^ (*n* = 1012^a^)	None	19%	29%	
	1–5	28%	35%	
	6–15	22%	19%	
	16–30	12%	10	
	More than 30	19%	8%	χ^2^(4) = 42.9, *p* < .001
Clinical position^b^ (*n* = 870^a^)	Resident	20%	30%	
	Specialist	29%	30%	
	Chief physician	51%	39%	χ^2^(4) = 19.1, *p* < .001

Reported are percentages of each category

^a^Not all totals reflect the full number of participants (*N* = 1018) because of missing entries

^b^Percentages refer to the number of responses within the given gender

^c^Percentages refer to the number of responses within the given country

### Measures

The questionnaire used in the current study consisted of 123 items and was part of the HOUPE II (Health and Organization among University Physicians in Seven European Countries) study (www.houpe.no). This questionnaire entailed self-reported answers from physicians employed at university hospitals across seven European countries (Austria, Hungary, Iceland, Italy, Norway, Sweden and the Netherlands). The current analysis includes only Austrian, Dutch and Swedish participants. Furthermore, only a selected set of seven variables was analysed.

#### Socio-demographic questions

Participants were asked for their self-reported gender (*female*, *male*), age and number of children. Age was reported in 5-year categories (*under 29* *years*, *30*–*34* *years*, *35*–*39* *years*, *40*–*44* *years*, *45*–*49* *years*, *50*–*54* *years*, *55*–*59* *years*, 60–64 *years*, *65* *years and older*). Physicians were asked to indicate whether they had *no children*, *one*, *two*, *three* or *more than three* children. The country in which the participants worked was noted in the corresponding questionnaire by the researchers.

#### Publication activity

To measure publication activity one question from the Physician Career Path Questionnaire—PCPQ (Fridner 2004) was used. Participants were asked, “How many peer-reviewed scientific articles have you authored or co-authored?” There were ordinal categorical response categories from which participants could select an answer (*no publications*, *1*–*5 publications*, *6*–*15 publications*, *16*–*30 publications*, *31*–*50 publications*, *51*–*100 publications*, *more than 100 publications*).

#### Working hours

Another open-ended question from the PCPQ (Fridner 2004) was used to assess the number of hours a physician worked at the university hospital per week.

#### Clinical position

A question concerning the physicians’ current clinical position was included in the analysis. Physicians were asked to select from three categories: “*resident doing specialist training*”, “*specialist/consultant/medical officer*”, “*chief physician/senior medical officer/director*”. These categories were chosen for the current study, because these categories were most equivalent among the participating countries. The category “*resident doing specialist training*” represented the lowest clinical position, whereby the position of “*chief physician/senior medical officer/director*” was the highest clinical position. Those participants who were unable to find their current clinical position among the three categories could choose *other*. The 84 (6.1%) participants who chose *other* were excluded from the analysis, because the clinical rank of these participants was unspecified.

### Procedure

The study was conducted as a cross-sectional online survey. All physicians at one university hospital in each of the three countries were invited by e-mail to participate. They were provided with a link to the online questionnaire of the HOUPE II study. Participation was anonymous and voluntary. Refusal to participate or failure to finish the questionnaire had no negative consequences. No reimbursement was offered.

In the Netherlands, the questionnaires were available online from April to June 2012. In Sweden, data collection also took place in 2012. For the Austrian sample, physicians could access the questionnaire for 10 weeks (from November 2012 to January 2013). Unfortunately, in the Austrian sample, the online system broke down several times. That could assumedly have negatively affected participants’ motivation to participate. The Austrian study was approved by the Ethics Committee of the Medical University of Innsbruck (UN 4807 316/4.11). The Swedish study was approved by the Regional Ethics Board (Stockholm; Number 04-913/2). According to the Dutch Medical Research Human Subjects Act (WMO 2015), the current study did not require formal approval, which was confirmed by the university’s medical ethics board. The study was conducted in accordance with the Declaration of Helsinki (World Medical Association 2013).

### Statistical analysis

Descriptive statistics of participants’ answers included the frequency and percentages of each given response. Gender differences for each of the four categorical variables (age, number of children, publication activity, clinical position) were analysed with the Chi Square test. Gender differences for the number of hours worked per week in the hospital were calculated with the *t* test. For these analyses the Statistical Package for the Social Sciences (SPSS) for Windows, version 24.0 (IBM Corp., Armonk, NY, USA), was used.

**Fig. 1:**
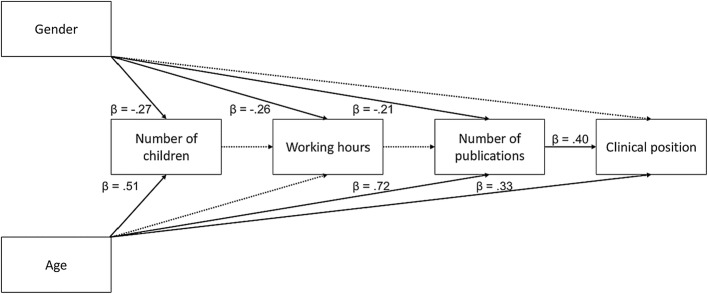
Path analysis model for predicting differences in clinical position. Standardised path coefficients are reported for significant interactions that are indicated by solid lines. Dotted lines indicate non-significant associations. Presented are the results for the Austrian sample

To determine associations between gender, parenthood, working hours, publication activity, age and clinical position, a structural equation model was calculated using MPlus, Version 8 (Muthén and Muthén 1998–2017). The model is depicted in Fig. 1. In the model for this manifest path analysis we viewed clinical position as the outcome of three variables. Namely, the model predicted clinical position by means of age, gender and publication activity. Publication activity in turn was predicted by working hours, whereby working hours were predicted by number of children. Age and gender were entered as predictive factors for every factor in the path model (Fig. 1).

The level of significance for all analyses was α = .05. All analyses were done separately for each of the three university hospitals.

## Results

### Austrian university hospital

#### Gender differences

Univariate group comparisons between women and men in the Austrian subsample revealed three gender differences (Table 1). Women reported having fewer children than did men and women indicated having fewer published articles than did men (Table 1). The last gender difference in the Austrian sample was found in the working hours. On average men worked more hours per week than did women (Table 1). The group comparison revealed no differences between women’s and men’s age and clinical position (Table 1).

#### Predictors of clinical position

In the structural path model (Fig. 1) clinical position was positively associated with the number of publications (β = .40, *p* < .001). Physicians with more publications were more likely to hold a higher clinical position than were physicians with fewer publications. Furthermore, age played a significant role (β = .33, *p* < .001) in predicting clinical position. Older physicians were more likely to hold higher clinical positions than were younger physicians. Gender was not directly associated with clinical position (β = − .02, *p* = .742). However, gender was indirectly significantly associated with clinical position through publication activity (β = − .08, 95%*CI* = − .14 to − .04, *p* = .003). The negative association between gender and publication activity (β = − .21, *p* < .001) indicated that women were likely to publish fewer articles than were men. This negatively impacted clinical position. The model explained 67.5% of the variance in clinical position.

Working hours were not associated with publication activity (β = .05, *p* = .422) nor were they associated with the number of children (β = − .02, *p* = .811). Gender was negatively associated with both variables, indicating that women were more likely to have fewer children (β = − .27, *p* < .001) and to work fewer hours per week (β = − .26, *p* = .006) than were men.

### Dutch university hospital

#### Gender differences

In the Dutch subsample men were significantly older than were women (Table 2). Univariate group comparisons between women and men in the Dutch sample revealed further gender differences for all other variables of interest. Women reported having fewer children than did men (Table 2). Women indicated having fewer published articles, and they worked fewer hours per week (Table 2) than did men. More men than women were found to hold higher clinical positions (Table 2).

#### Predictors of clinical position

**Fig. 2:**
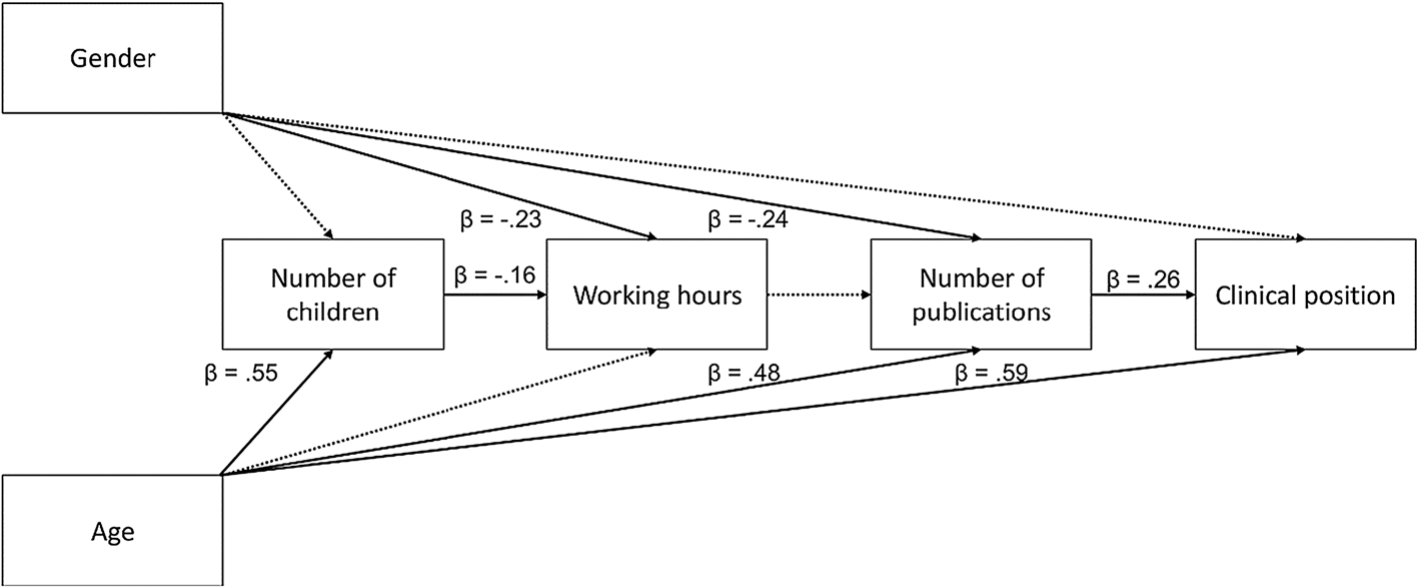
Path analysis model for predicting differences in clinical position. Standardised path coefficients are reported for significant interactions that are indicated by solid lines. Dotted lines indicate non-significant associations. Presented are the results for the Dutch sample

In contrast, the structural path model (Fig. 2) showed that clinical position was not directly predicted by gender (β = − .03, *p *= .601). In the Dutch subsample the effect of gender was indirectly seen in publication activity (β = − .06, 95%*CI* = − .12 to − .03, *p* = .002). Women were less likely to have published a large number of articles than were men (β = − .24, *p* < .001). Lesser publication activity in turn was associated with lower clinical position (β = .26, *p* < .001) (Fig. 2). Age positively predicted higher clinical position (β = .59, *p* < .001). The model explained 59.8% of the variance in clinical position.

Age further predicted a larger number of publications (β = .48, *p* < .001) and a larger number of children (β = .55, *p* < .001). Working hours were negatively associated with gender (β = − .23, *p* = .002) and number of children (β = − .16, *p* = .042). Thus, women and persons with more children were more likely to work fewer hours per week than were men and persons with fewer children. However, working hours were not associated with publication activity (β = .08, *p* = .149). The number of children was not associated with gender (β = − .07, *p* = .327) (Fig. 2).

### Swedish university hospital

#### Gender differences

The Swedish subsample showed univariate gender differences for four variables of interest. In the Swedish subsample men were older than women (Table 3). Men had more children than did women, they had more publications than did women and they held higher clinical positions than did women (Table 3). No gender difference was found in working hours (Table 3).

#### Predictors of clinical position

**Fig. 3:**
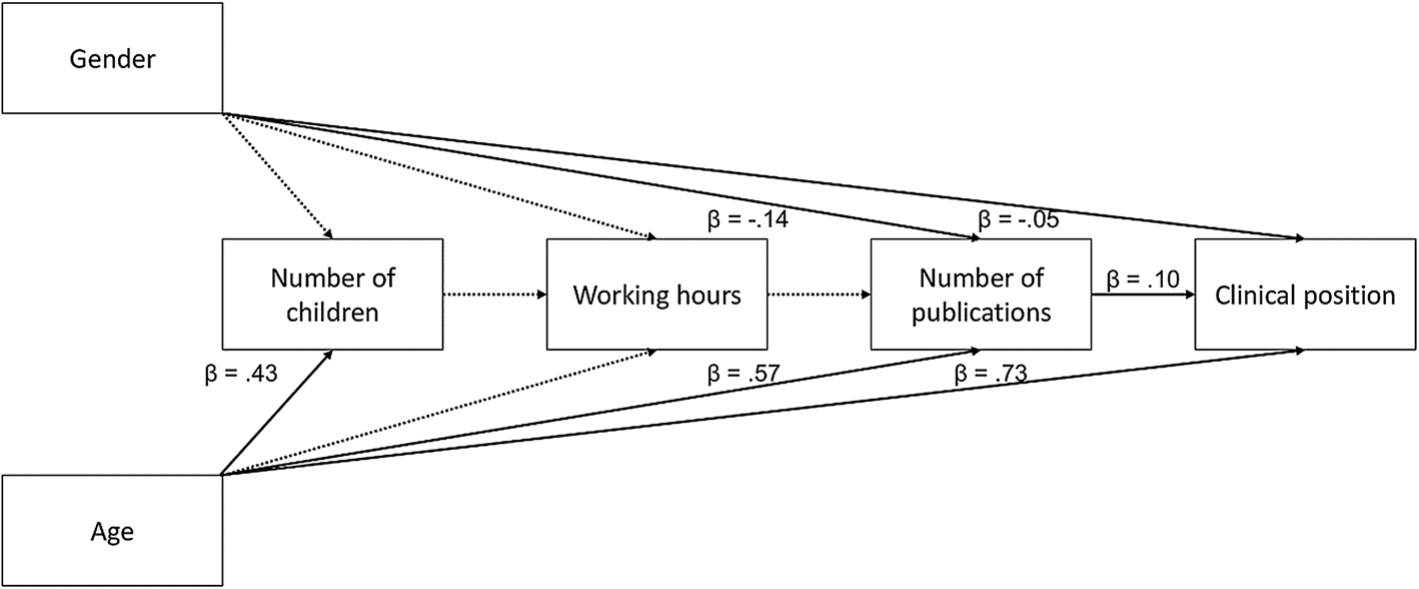
Path analysis model for predicting differences in clinical position. Standardised path coefficients are reported for significant interactions that are indicated by solid lines. Dotted lines indicate non-significant associations. Presented are the results for the Swedish sample

In the structural path model (Fig. 3) working hours were not associated with gender (β = .00, *p* = .910), nor with age (β = − .02, *p* = .536), nor with number of children (β = − .05, *p* = .184). Number of children was associated only with age (β = .43, *p* < .001), but not with gender (β = − .05, *p* = .081). Working hours did not predict the number of published articles (β = − .04, *p* = .119) (Fig. 3).

Both gender and age were directly associated with publication activity (β = − .14, *p* < .001 for gender and β = .57, *p* < .001 for age) and clinical position (β = − .05, *p* < .011 for gender and β = .73, *p* < .001 for age) (Fig. 3). Men and older physicians were more likely to publish more and were more likely to hold higher clinical positions than were women and younger physicians, respectively. The number of published articles in turn was predictive for higher clinical position (β = − .10, *p *< .001). As was the case in the other two path models, gender had an indirect effect on clinical position through publication activity (β = − .01, 95%*CI* = − .02 to − .01, *p* = 002). Age, number of publications and gender explained 63.4% of the variance in clinical position.

## Discussion

This study explored gender differences in clinical position among academic physicians at three different university hospitals in three European countries, namely Sweden, the Netherlands and Austria. We analyzed whether the number of children, working hours or publication activity could explain gender differences in physicians’ clinical position. It was expected that gender differences at these three university hospitals would differ in accordance with the countries’ Gender Equality Index (World Economic Forum 2015). We expected that in Sweden, a country known for its high gender equality, gender differences would be less prominent than in the Netherlands or Austria.

### Leaky pipeline

One conceptual framework to explain challenges in female academic physicians’ career progression is the “leaky pipeline” (Ahmad 2017; Etzkowitz et al. 2000; Goulden et al. 2011; National Research Council 1983), according to which starting a family and childbirth are two common factors influencing whether women stay on the academic track or leave it (e.g. Goulden et al. 2011). According to this concept, female academic physicians are more affected by family formation and childbirth in heterosexual couples because women are typically ascribed a larger share of household labor than are men (Berk 2012).

At all three participating universities Gender Strategies or Action Plans are enacted to ensure equal treatment of all employees and to banish discrimination at the work place. One important regulation includes parental leave policies (Luhan 2004; Maes et al. 2012; Rickegård 2017). Such policies ensure that people taking parental leave are not consequently discriminated or disadvantaged with regard to their position or job opportunities. Furthermore, these policies regulate access to adequate child-care facilities. All these policies work towards preventing the “leaky pipeline” from “leaking”. In the current study, at least the often cited hindrances of family formation and childbirth seem to have no effect on clinical position through working hours or publication activity. This finding is important because it indicates that the compulsory policies for specifically supporting gender equality and the balancing of work and “life” seem to be effective.

Another important finding of the study was that working hours were not associated with publication activity. Thus, the possibility to work more hours at the hospital may not result in higher publication rates. This may be caused by the need for an academic physician to divide working time among many tasks. Next to research and teaching, physicians need to tend to patients and they often need to tend to everyday administrative tasks (Thun et al. 2018; Wenger et al. 2017). It was found that among all those tasks female academic physicians as compared to male academic physicians are less likely to find time for research (e.g. Waljee et al. 2015). Given that publications are fundamental for job progression in academia (Leykum et al. 2011), women may be disadvantaged by allocating their working time to other tasks instead of doing research.

At all three university hospitals gender was found to influence clinical position through publication activity. This result adds to the literature that reports that female physicians are less likely to publish scientific articles than are male physicians (Burden et al. 2015; Eloy et al. 2013; Jagsi et al. 2006; Jena et al. 2015; Løvseth et al. 2014; Piper et al. 2016; Reed et al. 2011). Women could be helped in their job advancement that relies on publication activity by providing them with more role models, mentoring programs or encouraging them to publish beginning as early as residency (Edmunds et al. 2016; Forster 2000; Maes et al. 2012).

An additional factor that may explain the difference in publication activity between female and male physicians is the discrimination of work by female authors. It was found that people judge identical scientific texts differently solely on the basis of the author’s supposed gender (Goldberg 1968; Handley et al. 2015). Women’s publications are more likely to be underrated (Lerchenmueller and Sorenson 2018; Wennerås and Wold 1997) and women are disadvantaged in grant applications (Tamblyn et al. 2018). We concur with Holman, Stuart-Fox, and Hauser (2018) that efforts should be undertaken in academia to eliminate this discrimination of female authors’ work. Feminine and masculine stereotypes and other prejudices that are deeply rooted in the working environment and that lead to beliefs that women are less qualified for scientific work should be addressed, challenged and eliminated.

Hiring decisions should be made by a committee rather than individuals. During their decision-making process, members of such a committee should openly discuss gender equality concerns and possible underlying biases (Byington and Lee 2015). Additionally, in the promotion procedures greater weight should be given to non-research activities because of the found differences in publication activity and women’s strong involvement in non-research activities (e.g. Waljee et al. 2015).

### Direct effects of gender on clinical position

Even though of the three countries in this study (World Economic Forum 2015) Sweden is rated as the country with the highest rate of gender equality, it was in the Swedish sample that gender was seen to have a direct effect on clinical position, next to its indirect effect through publication activity. Female academic physicians were less likely to hold an academic administration or leadership position than were male academic physicians, even while controlling for publication activity.

One possible explanation for this unexpected finding may be offered by the “post-discrimination” rhetoric (Jenkins 2013, p. 81). At the Swedish university hospital academic physicians may believe that all regulations and policies in place ensure that women are not disadvantaged and therefore treated equally. Participants may have developed a “gender-blind” view (Sattari and Sandefur 2018, p. 6) and believe that gender no longer matters in their working environment. According to such a view, it is believed that the institution provides the needed policies and structures for a successful work-life balance to be possible, especially by providing the possibility for flexible working hours (Sattari and Sandefur 2018). However, such a standpoint shifts the task of providing the possibilities for a successful work-life balance from the institution to the individual. Management of work-life balance becomes a personal management task (Toffoletti and Starr 2016). Women may be especially challenged to meet such demands because of unequal allocation of household labor (Berk 2012). Achieving a work-life balance as an individual task may be viewed as an additional or impossible task, especially by female academic faculty (Toffoletti and Starr 2016), and an inability to manage work-life balance may be associated with personal failure and shame (Nash and Moore 2018).

Gender Strategies, Action Plans, or other policies in place at universities are an important step towards gender equality. However, as may be the case at the participating Swedish university hospital, actions need to be taken further, especially to challenge unconscious gender bias or “gender-blind” views (e.g. Maes et al. 2012). Clear communication about why Gender Strategies or Action Plans are put in place to specifically support women is also recommended in order to avoid resentment among people not affected by the specific offers (Sattari and Sandefur 2018). It is important to reinforce awareness for gender bias by conducting open conversations that reveal how gender (still) matters in the current working environment (Byington and Lee 2015; Sattari and Sandefur 2018).

### Strengths and limitations

Several limitations of the current study need to be addressed. First, the current study is a cross-sectional study assessing only one time point in the physicians’ career. Therefore, no conclusions about causality can be made and associations should not be misinterpreted as causal relationships. Furthermore, a longitudinal design would have allowed us to consider the gender distribution of medical students who commenced their medical education at a particular time point in relation to the gender distribution of those persons who ultimately reached leadership positions. The direct effect of gender on clinical position in the Swedish subsample may have been due to an unequal gender distribution among medical students a decade ago. An unequal gender distribution among medical students then could lead to an unequal gender distribution of persons who could potentially be considered for leadership positions now. However, among the Swedish medical students the number of women studying medicine or entering doctoral programs in medicine reached parity with the number of men in the past decade (e.g., Högskoleverket 2004, 2007, 2008). Nonetheless, additional studies with a longitudinal study design are needed to further clarify the causality of the found associations and to be able to follow the gender distribution across different career stages.

Second, different sample sizes among the three countries and different response rates could bias results. The Swedish sample was the largest and had the highest response rates. The other two countries, the Netherlands and Austria, had lower response rates. The Austrian sample showed an especially low response rate and a low participation number, which was attributed to the failings of the online system that broke down several times, assumedly affecting participants’ motivation to participate. Furthermore, the sample is not representative of all academic physicians in each of the three countries. Therefore, the results should be interpreted with caution and not be overgeneralized. Nevertheless, the current study offers a cross-country comparison of academic physicians’ working situation, in which gender differences in publication activity and clinical position become evident.

Third, a more accurate measure of publication activity than only number of publications would have shed more light on the publication success of the physicians. The employed retrospective measure of the number of published peer-reviewed scientific articles is prone to many biases. Nevertheless, gender differences showing lower publication rates became evident, comparable to studies that more stringently measure publication success (Holman et al. 2018). In summary, the current study showed that gender inequality in academic medicine has not been eliminated, also not at university hospitals in countries with high gender equality indexes.

## Conclusion

The current study explored gender differences in clinical position among academic physicians at three different university hospitals in three European countries, namely Sweden, the Netherlands and Austria. We analyzed whether the number of children, working hours or publications could explain gender differences in physicians’ clinical position.

The current study shows that parenthood did not influence working hours or, in turn, publication activity or clinical position. We posit that policies regulating parental leave and supporting gender equality in the balancing of work and “life” seem to be effective.

Despite the countries in this study having generally high rates of gender equality, gender equality has not yet been achieved in the three participating university hospitals. Women are still less likely than men to reach high academic positions at the university hospitals. Some but not all gender differences with regard to academic position are explained by the lower publication rates of female physicians as compared to male physicians. At the Swedish university hospital female academic physicians were less likely to hold a leadership position than were male academic physicians, even irrespective of publication activity. It is important to detect, challenge and eliminate underlying assumptions and feminine and masculine stereotypes among the work force. Only when gender is addressed as an important matter influencing the current working environment (Sattari and Sandefur 2018) can policies implemented at the universities or in the discipline of academic medicine to guarantee equality among women and men meaningfully help both women and men have a fair chance of equal promotion to top academic positions.
